# A Silicon Monoxide Lithium-Ion Battery Anode with Ultrahigh Areal Capacity

**DOI:** 10.1007/s40820-022-00790-z

**Published:** 2022-01-25

**Authors:** Jiang Zhong, Tao Wang, Lei Wang, Lele Peng, Shubin Fu, Meng Zhang, Jinhui Cao, Xiang Xu, Junfei Liang, Huilong Fei, Xidong Duan, Bingan Lu, Yiliu Wang, Jian Zhu, Xiangfeng Duan

**Affiliations:** 1 State Key Laboratory for Chemo/Biosensing and Chemometrics, College of Chemistry and Chemical Engineering, School of Physics and Electronics, Hunan Key Laboratory of Two-Dimensional Materials, Engineering Research Center of Advanced Catalysis of the Ministry of Education, Hunan University, Changsha, 410082 People’s Republic of China; 2 International Graduate School at Shenzhen, Tsinghua University, Shenzhen, 518057 People’s Republic of China; 3 Key Laboratory of Structures Dynamic Behavior and Control of the Ministry of Education, Key Laboratory of Smart Prevention and Mitigation of Civil Engineering Disasters of the Ministry of Industry and Information Technology, Harbin Institute of Technology, Harbin, 150090 People’s Republic of China; 4 School of Energy and Power Engineering, North University of China, Taiyuan, 030051 People’s Republic of China; 5 Department of Chemistry and Biochemistry, University of California, Los Angeles, CA 90095 USA

**Keywords:** Silicon monoxide, Large-sheet holey graphene, Lithium-ion batteries, High mass loading, Ultra-high areal capacity

## Abstract

Silicon monoxide (SiO) is an attractive anode material for next-generation lithium-ion batteries for its ultra-high theoretical capacity of 2680 mAh g^−1^. The studies to date have been limited to electrodes with a relatively low mass loading (< 3.5 mg cm^−2^), which has seriously restricted the areal capacity and its potential in practical devices. Maximizing areal capacity with such high-capacity materials is critical for capitalizing their potential in practical technologies. Herein, we report a monolithic three-dimensional (3D) large-sheet holey graphene framework/SiO (LHGF/SiO) composite for high-mass-loading electrode. By specifically using large-sheet holey graphene building blocks, we construct LHGF with super-elasticity and exceptional mechanical robustness, which is essential for accommodating the large volume change of SiO and ensuring the structure integrity even at ultrahigh mass loading. Additionally, the 3D porous graphene network structure in LHGF ensures excellent electron and ion transport. By systematically tailoring microstructure design, we show the LHGF/SiO anode with a mass loading of 44 mg cm^−2^ delivers a high areal capacity of 35.4 mAh cm^−2^ at a current of 8.8 mA cm^−2^ and retains a capacity of 10.6 mAh cm^−2^ at 17.6 mA cm^−2^, greatly exceeding those of the state-of-the-art commercial or research devices. Furthermore, we show an LHGF/SiO anode with an ultra-high mass loading of 94 mg cm^−2^ delivers an unprecedented areal capacity up to 140.8 mAh cm^−2^. The achievement of such high areal capacities marks a critical step toward realizing the full potential of high-capacity alloy-type electrode materials in practical lithium-ion batteries.

**Supplementary Information:**

The online version contains supplementary material available at 10.1007/s40820-022-00790-z.

## Highlights

- The large-sheet holey graphene framework/SiO (LHGF/SiO) composite displays notably high recoverable strain, suggesting considerably improved mechanical flexibility and robustness
- The LHGF/SiO anode with a mass loading of 44 mg cm^−2^ delivers a high areal capacity of 35.4 mAh cm^−2^ at current density of 8.8 mA cm^−2^ and retains a capacity of 10.6 mAh cm^−2^ at 17.6 mA cm^−2^
- The LHGF/SiO anode with an ultra-high mass loading of 94 mg cm^−2^ delivers an extraordinary areal capacity up to 140.8 mAh cm^−2^, about 1–2 order of magnitude higher than those in typical commercial devices

**Supplementary Information:**

The online version contains supplementary material available at 10.1007/s40820-022-00790-z.

## Graphical abstract

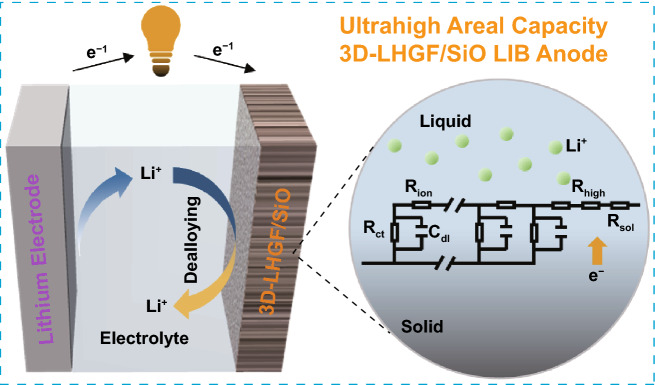

## Introduction

The graphite anode has shown tremendous success in commercial lithium-ion batteries (LIBs), but faces fundamental capacity limitations (372 mAh g^−1^) for the next generation of energy storage devices [1–3]. To this end, the search for electrode materials with higher specific capacity is drawing increasing attention. Silicon monoxide (SiO) represents an attractive anode material for high-energy density LIBs for its high theoretical capacity (2680 mAh g^−1^, assuming all the Si in SiO can be converted into Li_4.4_Si during lithiation) [3, 4]. However, SiO anodes are generally plagued with inherently poor charge (electrons and ions) transport properties and the large volume change that leads to rapid pulverization during cycling, both of which limit the achievable mass loading and contribute to poor cycling performance with rapidly fading capacity [5–7].

Considerable efforts have been exerted in adopting SiO into LIBs anode to realize high reversible capacity [8, 9]. However, the studies to date have been largely limited to proof-of-concept demonstrations with a relatively low mass loading of SiO (0.8–3.5 mg cm^−2^), which limits the overall areal capacity and poses a serious challenge for practical technologies [10, 11]. In today’s LIBs, the graphite anodes with mass loading of ~ 10 mg cm^−2^ typically deliver an areal capacity of ~ 3 mAh cm^−2^ [12, 13]. For next-generation LIBs, the areal capacity should reach up to ~ 6 mAh cm^−2^ in order to minimize the mass contribution associated with electrode area-dependent passive components (e.g., current collectors and separators) [14, 15]. To this end, it is highly desirable to implement high capacity materials in high mass loading electrodes [14]. The fabrication of such high-mass-loading electrodes can not only simplify manufacture steps to lower the cost but also boost the overall cell-level energy density [15].

However, there are considerable fundamental challenges in implementing high-capacity alloy-type materials with high mass loading using the conventional slurry-based electrode architecture. First, the high-capacity alloy-type electrodes typically feature a very large volume change during the charge/discharge process. When implemented in high mass loading, such large volume change could result in serious physical disintegration, pulverization and delamination from the current collector (Cu or Al foil). Second, the implementation of high-capacity electrode materials with high mass loading requires an unusually high charge transport capability for both electrons and ions to supply sufficient charge to fully utilize the storage capacity of the high capacity-material, which is beyond the limit of current slurry electrode design [10, 15]. Therefore, the implementation of high capacity electrode in high mass loading electrode remains a persistent challenge for the field, and a critical roadblock towards the realization of the full potential of high-capacity materials.

Here, we report a monolithic three-dimensional (3D) large-sheet holey graphene framework/SiO (LHGF/SiO) composite with several distinguished advantages for ultrahigh areal capacity electrode. First, the assembly of 3D hierarchical porous structure with large sheet graphene and extended-interaction ensures exceptional mechanical robustness and flexibility to accommodate the large volume change of SiO and ensures the structure integrity even at ultrahigh mass loading. Second, the continuous graphene network structure ensures excellent electron conductivity, while the hierarchical 3D porous structure with fully interconnected microscale and nanoscale channels greatly promote the ion transport, both of which are critical for efficient charge delivery necessary for ultra-high mass loading. Together, we show the LHGF/SiO electrodes with an ultra-high mass loading of 94 mg cm^−2^ deliver an ultra-high reversible areal capacity up to 140.8 mAh cm^−2^, and the full-cells energy density reaches up to 393 Wh kg^−1^, greatly exceeding those in the advanced research or commercial devices. The study represents a critical step forward toward implementing high-capacity SiO-based electrode materials in practical devices.

## Experiment Section

### Preparation of Large-Sheet Holey Graphene Aqueous Solution

Large-sheet graphene oxide (LGO) was prepared by oxidation of crystalline flake graphite (50 mesh; XFNANO Tech. Co., Ltd, Nanjing, China) following a modified Hummers’ method, and the large-sheet holey graphene oxide (LHGO) was prepared by the previous method [12, 16]. Briefly, 50 mL of 2 mg mL^−1^ LGO aqueous dispersion was mixed with 5 mL 30% H_2_O_2_ aqueous solution and then heated at 90 ℃ under stirring for 2 h. According to the previous studies, the oxidative-etching process initiates from the chemically more active oxygenic defect sites and propagates in the basal plane of LGO to form increasingly larger pores with increasing etching time. Then, the in-plane pores in the holey graphene sheet function as ion transport shortcuts in the hierarchical porous structure to facilitate rapid ion transport throughout the entire 3D electrode and greatly improve ion access to the surface of the SiO [12, 16]. The as-prepared LHGO was purified by repetitive centrifuging and washed to remove the residual H_2_O_2_ and then re-dispersed in deionized (DI) water.

### Preparation of LHGF/SiO Composite

The LHGF/SiO composite anode was prepared using a two-step process. Firstly, the large-sheet graphene/SiO (LG/SiO) composite was prepared by mixing SiO powder (99.9%, Sigma-Aldrich) and LGO solution with a mass ratio of 2:1. The product was freeze-dried (KRSYQ-10A, Changsha China) and annealed at 850 ℃ under argon flow for 4 h. Secondly, the LG/SiO sample was dispersed into HGO with a mass ratio of 5:2 and then experienced a thorough magnetic stirring. After that, excess ascorbic acid (VC) (*m*_VC_:*m*_LHGO_, 4:1) was added into this aqueous mixture and heated at 90 ℃ for 6 h to synthesize LHGF/SiO composite hydrogel with 75% SiO (LHGF/SiO-75%). The LHGF/SiO composite hydrogel with 50% SiO (LHGF/SiO-50%) was obtained using the same method by controlling the *m*_G/SiO_:*m*_LHGO_ as 2:1. The as-prepared LHGF/SiO composites were washed by DI water for three times to remove needless impurities. After freeze-drying, the samples were annealed at 850 ℃ under argon flow for 4 h. As a comparison, the large-sheet graphene framework/SiO (LGF/SiO) composite without holey structure was obtained using the same two-step process by replacing LHGO with LGO aqueous solution.

### Preparation of Pure Slurry SiO Electrode

The SiO powder, conductive carbon and polyvinylidene fluoride (PVDF) with a mass ratio of 20:3:4 were dispersed in a mixed N-methyl pyrrolidone (1.2 mL) under magnetic stirring. Then, the slurry was painted onto a Cu foil and vacuum-dried at 60 ℃ for 24 h as anode. The mass loading of the SiO electrode was about 11 mg cm^−2^.

### Material Characterization

The morphology and structure of materials were characterized by scanning electron microscopy (SEM, Mira3 TESCAN, Czech Republic), transmission electron microscopy (TEM, Titan S/TEM FEI, America), X-ray diffraction patterns (XRD, SmartLab 3 kW Rigaku, Japan), inVia-reflex confocal Raman spectrometer with a 633-nm laser as the excitation source (Renishaw, UK). The mechanical properties of the HGF/SiO and LHGF/SiO were studied using an Instron 3365 universal testing machine with 100-N load cells at a strain rate of 10 mm min^−1^ (Instron, 3365, America). Nitrogen adsorption–desorption isotherm at 77 K was measured on an ASAP 2020 absorption analyzer (Micromeritics, America). The specific surface area and the pore size distribution of the samples were deduced using the Brunauer–Emmett–Teller (BET) and the Barrett–Joyner–Halenda (BJH) analysis method, respectively. Thermogravimetric analysis (TGA, HTG-1, Beijing China) was conducted in the air atmosphere from room temperature to 800 ℃ at a heating rate of 10 ℃ min^−1^.

### Electrochemical Characterization

The free-standing LGF/SiO and LHGF/SiO composites were mechanically compressed and served as the working electrode without any binder or conductive additive with lithium metal as counter and reference electrode to assemble 2032-type half-cell in an argon-filled glove box under water and oxygen content below 0.5 ppm. The lithium hexafluorophosphate (LiPF_6_) dissolved in a mixture of ethylene carbonate (EC), dimethyl carbonate (DMC), diethyl carbonate (DEC) and fluoroethylene carbonate (FEC) was used as the electrolyte (1.0 M LiPF_6_ in EC/DMC/DEC + 5% FEC, *V*_EC_:*V*_DMC_:*V*_DEC_ = 1:1:1, Nanjing Mojiesi Energy Tech., Co., Ltd., Nanjing, China). The typical areal mass loadings of the electrode materials were controlled to 11, 21, and 44 mg cm^−2^ for the studies of mass loading dependence. The compacted density of the electrodes is around 1.3 g cm^−3^. The galvanostatic charge/discharge cycling and alternating-current impedance tester (in a frequency range between 100 kHz and 0.01 Hz at potentiostatic signal amplitude of 10 mV) were conducted in a multichannel battery testing system (LAND CT2001A, LANDTE Co., Wuhan, China) and Vertex.One.EIS electrochemical station (IVIUM Technologies BV, Eindhoven, Netherlands), respectively. The simulation of the experimental impedance was conducted with IVIUM software. The equivalent circuit for the fitting of impedance spectra at the SOC of 0% was conducted by the generalized finite length Warburg element (W) for descriptive purposes [17, 18].

## Results and Discussion

### Synthesis and Characterization of the Hierarchically Porous Composites

**Fig. 1:**
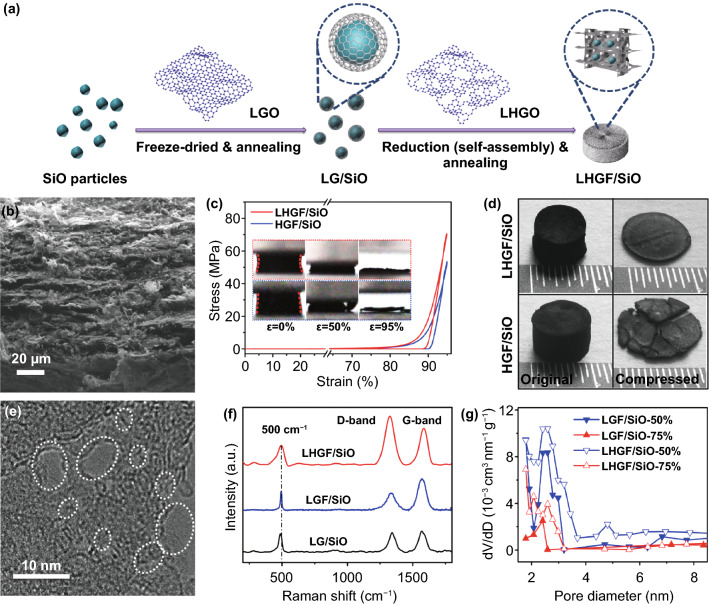
Illustration of the two-step process flow to prepare 3D hierarchically porous composite architecture and structural characterization of LHGF/SiO composite. **a** Illustration of preparation process of LHGF/SiO composite. **b** Cross-sectional SEM image of the compressed 3D composite. **c** Uniaxial compression of LHGF/SiO and HGF/SiO with strain up to 95%. Inset: experimental snapshots of the LHGF/SiO and HGF/SiO composite during uniaxial compression. **d** The photograph showing corresponding structure before and after mechanical compression (95% stress). **e** TEM image of graphene sheet with tailored pores. **f** Raman spectra of LHGF/SiO, LGF/SiO and LG/SiO composites. **g** Comparison of BJH pore size distribution for LHGF/SiO and LGF/SiO composites

The LHGF/SiO composites were prepared as a freestanding electrode via typical freeze-drying, annealing, reduction (self-assembly) and another high-temperature annealing process (Fig. 1a). Scanning and transmission electron microscopy studies show SiO particles are uniformly distributed within 3D graphene network structure and with an average size around 3 μm (Figs. 1b and S1). Since the mechanical flexibility is essential for accommodating the large volume change and retaining the structure integrity at high mass loading (thick electrode), we have carefully tailored the starting graphene sheets to ensure extraordinary mechanical robustness.

Previous efforts on HGF typically use relatively small graphene sheets (0.5–2.0 μm in lateral dimension), which often shows insufficient mechanical robustness for ultrahigh mass loading composite electrode [19, 20]. To address this challenge, we specifically used large-sheet (30–40 μm in lateral dimension) holey graphene oxide (LHGO) as the starting materials for constructing large-sheet holey graphene framework (LHGF) with considerably increased mechanical flexibility and robustness (see Method for details) [20–22]. The LHGF features the well-interconnected scaffolds with strong extended *π*–*π* interactions among large graphene sheets to endow super-elasticity and exceptional mechanical robustness [20–22]. We investigated the mechanical properties of HGF/SiO and LHGF/SiO with uniaxial quasi-static compression. As shown in Fig. 1c, the stress–strain curve demonstrates LHGF/SiO exhibits an extremely large compressibility with strain up to 95% and corresponding stress of 70 MPa. The compressed stress and recoverable strain of LHGF/SiO is obviously higher than that of the HGF/SiO, indicating considerably improved mechanical flexibility and robustness of LHGF due to larger stacking interfaces with robust *π*–*π* interactions. From the viewpoint of the Young’s modulus, in mathematics, the Young’s modulus is calculated by Eq. (1):

1 $$E = \frac{\sigma }{\varepsilon }$$

where *σ* is the uniaxial stress and ε is the strain [20, 21]. The LHGF/SiO (1.5 MPa) exhibited higher Young’s modulus than the HGF/SiO (1.2 MPa) indicating that the designed hierarchical LHGF/SiO can easily resist expansion of SiO under charge/discharge cycling. Meanwhile, the tensile strength test for composites is based on Eq. (2):

2 $$\sigma_{b} = \frac{F}{S}$$

where *F* is the maximal uniaxial stress and *S* is cross-sectional area of the sample [20, 21]. The *σ*_*b*_ value of LHGF/SiO is 4 kPa higher than that of HGF/SiO (Fig. S2), which demonstrates the monolithic LHGF is hard to be deformed, thus suggesting the positive effect to resist volume change of SiO [20–22]. In addition, the volume variation observed at lithiation results from the partial accommodation of particle expansion by the electrode porosity. Actually, compared to the HGF/SiO, the cross-sectional SEM images of LHGF/SiO illustrate that the cellular walls of LHGF are quite stable even though volume expansion of the SiO can reach ~ 200% (average size around 6 μm) during discharge process (Figs. S3 and S4).

It is interesting to note that the cylindrical LHGF/SiO sample demonstrates typical hyperboloid-shaped shrinkage in the macrostructure configuration under longitudinally applied compression (upper inset in Fig. 1c), suggesting a peculiar negative Poisson’s ratio (PR) behavior, which has been observed in similar graphene aerogel structure and is beneficial for retaining the structural stability upon compression [20, 21]. In contrast, the HGF/SiO sample shows slightly biconvex (indicating typical positive PR behaviors [20]) instead of hyperboloid-shaped shrinkage under longitudinally applied compression, with apparent cracks and some pieces breaking off from the cylindrical composite (low insets in Fig. 1c). Indeed, when a thick composite (with equivalent mass loading of ~ 30 mg cm^−2^) was compressed for 95% for battery assembly, the LHGF/SiO composite well maintains the structural integrity (upper images in Fig. 1d), while the HGF/SiO composite with a similar mass loading shows apparent pulverization (lower images in Fig. 1d) cannot be used for battery assembly. Such an elaborate mechanical design with large sheet holey graphene is essential for retaining the structural stability at an ultrahigh mass loading.

For comparison purpose in battery performance, we have prepared LHGF/SiO composite as well as the non-holey counterpart (LGF/SiO) using a similar process, with two mass fractions of SiO of 50 and 75 wt% (Fig. S5). The as-synthesized LHGF/SiO before annealing is amorphous as indicated by XRD studies (Fig. S6) and remains amorphous after calcination at 850 ℃ in argon [23]. TEM and selected area electron diffraction (SAED) studies also confirm the micrometer-scale SiO particles are amorphous (Fig. S1d) [24, 25]. High-resolution TEM studies confirm the porous structure of holey graphene sheets obtained by etching in H_2_O_2_ solution, with pore sizes of about 2–4 nm (Fig. 1e). The in-plane pores in the holey graphene sheet function as ion transport shortcuts in the hierarchical porous structure to facilitate rapid ion transport throughout the entire 3D electrode and greatly improve ion access to the surface of the SiO extending the reaction time which would lead to a more aggressive etching of LGO, enlarging the pore size. The Raman spectroscopy shows the D (~ 1340 cm^−1^) and G (~ 1590 cm^−1^) bands of graphene, and a characteristic band of SiO (500 cm^−1^) (Fig. 1f) [26]. The LHGF/SiO shows more pronounced D-band signal than that of LGF/SiO composite due to more disorders and defects in the nanoporous structure of the holey graphene sheets [27]. The N_2_ adsorption/desorption isotherms indicate the four different composites with various cumulative volumes of pores (Fig. S7). The total surface area calculated by BET method decreases from 52 to 28 m^2^ g^−1^ with the increase in the weight percentages of SiO for LHGF/SiO-50% and LHGF/SiO-75%. Similarly, the surface area of LGF/SiO-50% and LGF/SiO-75% decreases from 39 to 16 m^2^ g^−1^ (Table S1). Meanwhile, the Barrett–Joyner–Halenda (BJH) analysis reveals that the total pore quantity in the LHGF/SiO is more than twice of that in the LGF/SiO composite (Fig. 1g).

### Evolution of Kinetic Properties and Electrochemical Characteristics with Porosity

**Fig. 2:**
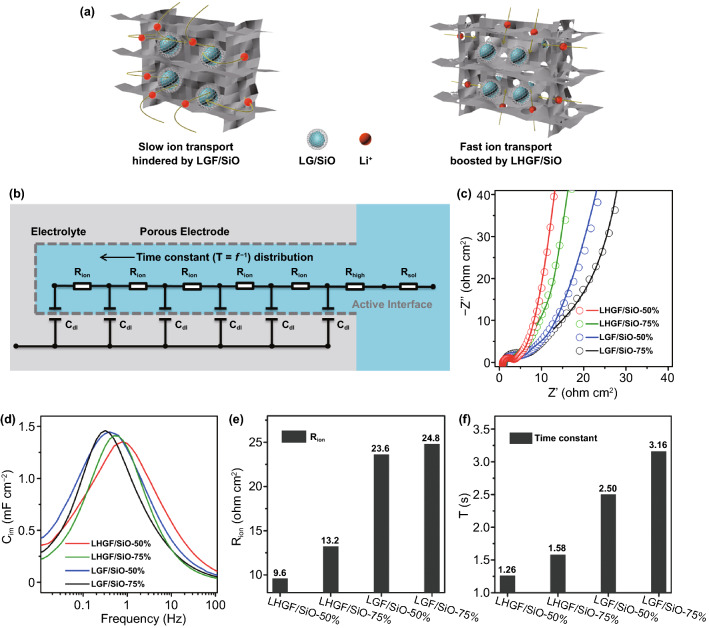
Evolution of kinetic properties and electrochemical characteristics with porosity. **a** Li^+^ transport schematic diagram of LHGF/SiO and LGF/SiO electrode. **b** Schematic representations of porous electrode structure and the equivalent circuit models Non-faradaic process at unlithiated state. **c** Nyquist plots for composites by using a symmetric cell with two identical electrodes (11 mg cm^−2^). The symbols and solid lines represent the experimental and simulation results, respectively. **d** The imaginary part of capacitance as a function of the frequency for various composite electrodes at a mass loading of 11 mg cm^−2^. **e** The ionic resistance (*R*_ion_) of different composite electrodes. **f** The time constant (T) as a function of different composite electrodes

The hierarchical porous structure of LHGF/SiO provides a rapid interpenetrating pathway for efficient transport of both electrons and ions [28]. In particular, with the unique design of LHGF architecture, Li^+^ can rapidly pass through the in-plane nanopores in the holey graphene, thus greatly shortening its conduction pathway compared with non-holey graphene framework, leading to more efficient ion transport (Fig. 2a) [12, 29]. To this end, we have conducted the electrochemical impedance spectroscopy (EIS) measurements using a symmetric cell with two identical electrodes to probe the effect of structural features on the ion transport kinetics. Compared to the conventional EIS tests of half-cells with the issue of overlapping profiles for the anode and cathode, symmetric cells using identical electrodes were adopted to decouple the internal resistance. Electrochemical processes in porous electrodes include the electrolyte bulk resistance (*R*_sol_), the ionic resistance in pores (*R*_ion_), an electric double layer at the electrode/electrolyte interface (*C*_dl_), and charge transfer resistance (*R*_high_, the *R*_high_ is equaled to *R*_ct_ in the impedance theory for pores based on the transmission line model (TLM)). All of them can be interpreted by the TLM (Fig. 2b) [30].

The Nyquist plots describe a non-faradaic process when the state of charge (SOC) is at 0% (Fig. 2c). Mathematically, the real axis projection length values are defined as *R*_ion_/3, where *R*_ion_ is the ionic resistance of the electrolyte-filled pores inside the 3D electrode architecture (see Supplementary Text for more details), which is a determining factor for the rate capability (Fig. S8) [31]. This projection is defined as *R*_ion_/3, as derived from a TLM for cylindrical pores. The gradual changes in projection length values for the different electrodes show a decrease in ionic resistance (*R*_ion_) from 24.8 to 9.6 Ω cm^2^, with increasing pore size in the LHGF scaffold [30]. Significantly, the *R*_ion_ values in the holey LHGF/SiO composites are notably lower than that in the non-holey LGF/SiO composites (Fig. 2e), highlighting the critical role of the in-plane pores in facilitating the efficient ion transport in the 3D composites. Previous reports have shown *R*_ion_ is the major factor influencing the electrochemical properties, particularly in thick electrodes [29, 31]. We have also used the TLM to simulate the EIS and obtained resistance values for various electrodes (Table S2).

Ion transfer behavior during the non-faradaic process can be also evaluated by complex capacitances (see Supplementary Text). In the theory of EIS, the imaginary capacitance (*C*_im_) displays a normal distribution curve with regard to frequency (Fig. 2d). The reciprocal of the peak frequency corresponds to the relaxation time constant of the electric double-layer, which is an important parameter in characterizing the ion-transfer rate or responsiveness time [29–31]. Again, the optimized holey LHGF/SiO composites show a much shorter time constant (1.26 and 1.58 s for LHGF/SiO-50% and LHGF/SiO-75%, respectively) than that of non-holey composites (2.50 and 3.16 s for LGF/SiO-50% and LGF/SiO-75%, respectively) (Fig. 2f) [29, 31]. Overall, in this system, these studies illustrate that the ion transport kinetics can be greatly boosted by tailoring the in-plane pores in the large holey graphene sheets that form the 3D graphene scaffold [31].

In the literature, the following lithiation mechanism of SiO has been proposed to occur during the charge/discharge process that has been widely accepted [3, 31]:

3 $${\text{SiO}}\mathop{\longrightarrow}\limits^{{{\text{Li}}}}\frac{1}{4}{\text{Li}}_{4} {\text{SiO}}_{4} + \frac{3}{4}{\text{Si}}\quad \left( {{\text{irreversible}}} \right)$$

4 $$\frac{1}{4}{\text{Li}}_{4} {\text{SiO}}_{4} + \frac{3}{4}{\text{Si}}\overset {{\text{Li}}} \longleftrightarrow \frac{1}{4}{\text{Li}}_{4} {\text{SiO}}_{4} + \frac{3}{4}{\text{Li}}_{3.75} {\text{Si}}\quad \left( {{\text{reversible}}} \right)$$

During reaction (3), an active/inactive composite phase is formed, in which the active Si phase can be reversibly cycled within the inactive Li_4_SiO_4_ matrix. Here, Li_4_SiO_4_ has been reported to be a Li-ion conductor [32]. And the active phase can combine with approximate four times Li-ions to deliver high capacity (2680 mAh g^−1^) in theory [3]. All of these factors permit the ultrahigh mass loading and thick electrode performance.

To elucidate the roles of SiO and graphene in the LHGF/SiO for Li storage, we used cyclic voltammetry experiments to analyze the capacity contribution mainly from the diffusion-controlled process (Fig. S9 and supplementary text) [12]. For scan rates from 0.1 to 0.5 mV s^−1^, *b* ~ 0.24 indicates that charge storage is largely controlled by semi-infinite diffusion, which is beneficial to high-energy density devices (Fig. S9b–c). The data for the LHGF/SiO-75% electrode and the fast surface-controlled process can only be exceeded up to 20 mV s^−1^ (Fig. S9d). Generally, inside of the hierarchical 3D structure with a large number of new surfaces would generate several electric double-layers during the charge/discharge process. We used microparticles (not nanoparticles) to fill in 3D structure interlamination to separate the electric double-layer structure. Therefore, the charge storage is predominantly controlled by diffusion, which is beneficial to high-energy density devices. Together, these studies further establish that LHGF with optimized in-plane pores is essential for electrolyte transport throughout the entire electrode for the full utilization of the active materials and realization of high areal capacity properties at high levels of mass loading [12, 13].

### Effects of Mass Loading on Electrochemical Characteristics

**Fig. 3:**
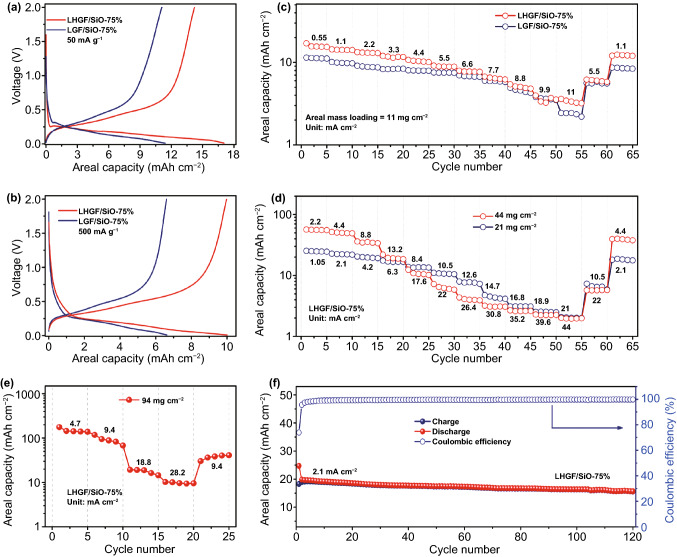
Effects of mass loading on electrochemical characteristics. Galvanostatic charge/discharge curves of two type electrodes at **a** 50 mA g^−1^ and **b** 500 mA g^−1^. The mass loading is 11 mg cm^−2^. **c** Rate performances of LHGF/SiO-75% and LGF/SiO-75% electrode at the same mass loading of 11 mg cm^−2^. **d** Rate performance of LHGF/SiO-75% electrode at the mass loadings of 21 and 44 mg cm^−2^. **e** Rate performance of LHGF/SiO-75% electrode at the mass loading of 94 mg cm^−2^. **f** Cycling performance of LHGF/SiO electrode with the mass loading of 21 mg cm^−2^ at a current density of 2.1 mA cm^−2^

With well-designed 3D hierarchical structure of LHGF/SiO, superior electrochemical performance can be obtained. A series of galvanostatic charge/discharge curves of LGF/SiO and LHGF/SiO with different SiO ratio and current densities at the mass loading of 11 mg cm^−2^ were investigated. The results indicate that the LHGF/SiO-75% electrode presents higher areal capacity than that of LGF/SiO-75%, LHGF/SiO-50% and LGF/SiO-50% at given current densities (Fig. 3a, b and S10). The optimized LHGF/SiO-75% electrode shows a relatively high areal capacity at different current densities (Fig. 3c). For example, the LHGF/SiO-75% electrode with mass loading of 11 mg cm^−2^ presents high areal capacity of 13.3 mAh cm^−2^ at a current density of 2.2 mA cm^−2^, which is 50% higher than that of the LGF/SiO-75% electrode (8.8 mAh cm^−2^). Compared with LGF/SiO electrode, the LHGF/SiO electrode clearly shows an enhanced Li^+^ storage property at a given rate, which is attributed to the in-plane nanopores on graphene sheets that provide adequate ions transfer channels to reduce the internal resistance and the associated potential drop. As current density is increased to a higher level of ~ 5.5 mA cm^−2^, the areal capacity fades rapidly, which is mainly limited by the penetration depth of ionic current in thick electrode and signifies the ion transport limit of this electrode architecture design [33]. In other words, the LHGF/SiO-75% and LGF/SiO-75% display similar transportation performance of electron at high current densities, leading to slightly different between LHGF/SiO-75% and LGF/SiO-75% in rate performance, especially at a current density of 6.6–9.9 mA cm^−2^. After that, as an important parameter of flexible device, the folding endurance was also evaluated by typical voltage profiles of the flexible battery devices after being bended from vertical direction for 20 times. No remarkable change of the charge/discharge profiles is observed from 2nd to 50th continuous cycles, indicating outstanding mechanical flexibility (Fig. S11).

Subsequently, three levels of mass loadings were investigated, corresponding to practical levels of loading (11 mg cm^−2^), and representative of future high levels of loading of 21 and 44 mg cm^−2^. The LHGF/SiO-75% electrode shows a significant increase in capacity with increasing mass loading (Fig. 3d). Impressively, at the mass loading of 44 mg cm^−2^, the LHGF/SiO-75% delivers a high areal capacity of 35.4 mAh cm^−2^ at a current of 8.8 mA cm^−2^, and retains a capacity of 10.6 mAh cm^−2^ at the ultra-high current of 17.6 mA cm^−2^. Both the areal capacity and the current density are much higher than those of the state-of-the-art commercial anodes (~ 3.5 mA cm^−2^ and 4.0 mAh cm^−2^). To further explore ultimate limit of the areal mass loading, battery performance of LHGF/SiO-75% electrode with a mass loading of 94 mg cm^−2^ is investigated (Fig. 3e). Significantly, the electrode exhibits an exceptional areal capacity of 140.8 mAh cm^−2^ under a current density of 4.7 mA cm^−2^, and a high areal capacity of 10.3 mAh cm^−2^ even at an ultra-high current density up to 28.2 mA cm^−2^.

These results indicate that the highly interconnected interpenetrating network in the LHGF promotes the rapid transfer of ions/electrons, and thus enables ultra-high areal capacity at various current densities well beyond the current state of the art [34]. With the monolithic 3D architecture, the LHGF shows excellent mechanical robustness to accommodate the repeated volume change during charge/discharge process and thus ensures cycling stability. The LHGF/SiO-75% electrode could deliver a reversible areal capacity of 15.6 mAh cm^−2^ at a mass loading of 21 mg cm^−2^, and a capacity retention of 79% at the current density of 2.1 mA cm^−2^ after 120 cycles, representing an excellent cycling stability among high areal capacity electrodes (Fig. 3f). Besides, the initial Coulombic efficiency (ICE) of LHGF/SiO-75% is as high as 74%.

### Effect of Mechanical Reinforcement on Achievable Thickness

**Fig. 4:**
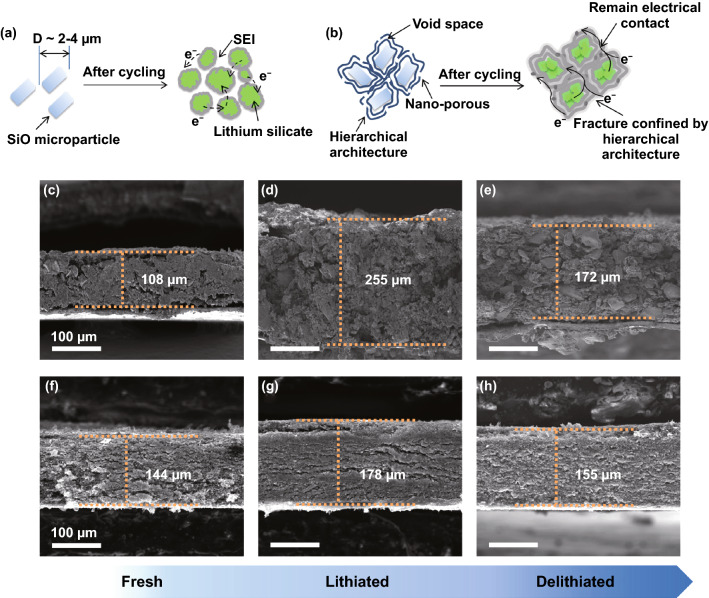
The electrode morphology characterization and structure analysis during the charge/discharge state. **a**, **b** Schematic illustration of the structural change in conventional slurry SiO electrode and LHGF/SiO electrode after cycling. Cross-sectional SEM images of conventional slurry SiO electrode (containing Cu current collector) at **c** fresh, **d** lithiated and **e** delithiated states. Cross-sectional SEM images of LHGF/SiO electrode at **f** fresh, **g** lithiated and **h** delithiated states

To further probe the impact of LHGF in accommodating and buffering the volume changes, we have analyzed the electrode structure after charge and discharge process. For a typical SiO-based slurry electrode, the volume expansion and contraction during the charge/discharge process could cause notable irrecoverable pulverization, physical disintegration, generating considerable porosity, leading to macroscopic volume expansion (Fig. 4a). Indeed, the cross-sectional SEM images of a conventional slurry SiO electrode clearly show extensive pore/crack formation along with a thickness increase from 108 to 255 µm after the first lithiation process (Fig. 4c, d), corresponding to ~ 126% volume expansion. The thickness shrinks back to 172 µm after delithiation (Fig. 4e), indicating ~ 60% irreversible volume change. Such a large change would inevitably lead to electrical disconnection and rapidly capacity fading.

In contrast, with encapsulation by the mechanically strong monolithic LHGF framework, the volume change in SiO particles in LHGF/SiO electrode is microscopically accommodated by the built-in porosity within the 3D LHGF, with little impact to the overall electrode structure, and little volume change during the lithiation and delithiation process (Fig. 4b). In particular, the LHGF/SiO electrode shows a relatively small thickness increase from 144 to 178 µm after lithiation (Fig. 4f, g). After delithiation process, the thickness decreased back to 155 µm (Fig. 4h), with only 8% increase over the original electrode, confirming the physical integrity of the electrode are well retained in LHGF/SiO, which is essential for ensuring the electrical integrity to stabilize cycling performance of the electrode.

### Implications of High Mass Loading

**Fig. 5:**
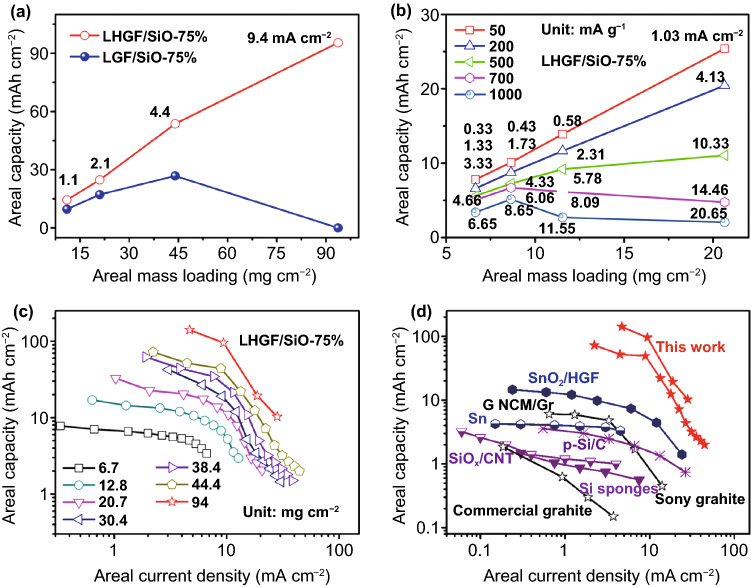
The scaling relationship for increasing mass loadings and comparison of energy storage performance metrics for various anode materials. **a** Dependence of areal capacity on mass loading at 100 mA g^−1^ for the LHGF/SiO-75% and LGF/SiO-75% electrodes. **b** The areal capacity versus mass loading for the LHGF/SiO-75% electrodes at various current densities. **c** The areal capacity versus current density for the LHGF/SiO-75% electrodes at various mass loadings. **d** The comparison of areal performance metrics of LHGF/SiO-75% electrode with various research anodes

In principle, the areal capacity scales linearly with mass loading in the ideal case. However, the inherent charge transfer limitation generally results in a nonlinear relationship in most electrode design, particularly at high mass loading or high rate when the charge transport limitation worsens [35]. Figure 5a compares the areal capacity versus mass loading for LHGF/SiO-75% and LGF/SiO-75% electrode at the same current density of 100 mA g^−1^. Importantly, the areal capacity of LHGF/SiO-75% electrode shows a nearly linear scaling relationship with mass loading, and the highest areal capacity reaching up to 140.8 mAh cm^−2^ is achieved at the highest mass loading of 94 mg cm^−2^. It is noted the slope decreases slightly at high mass loading regime. In contrast, the LGF/SiO-75% electrode shows much slow increase and the areal capacity peak at 26.8 mAh cm^−2^ at the mass loading is 44 mg cm^−2^. Further increasing the mass loading leads to an even lower areal capacity, indicating charge transport limitation plays a dominant role in thick electrode. Such capacity fading is attributed to concentration effects at high current density (from 8.09 to 11.55 mA cm^−2^) [12]. The resulted concentration gradient further restricts rapid charges transfer, resulting in capacity decrease [12, 36, 37].

Figure 5b shows the dependence of the areal capacity of LHGF/SiO-75% electrode at different current densities. When the current density of LHGF/SiO-75% is beyond 2–5 mA cm^−2^, the areal capacity starts to deviate from linearity and eventually reach a plateau at 11.0 mg cm^−2^. The plateau-like behavior at ~ 10 mA cm^−2^ has been interpreted as reaching a limiting condition, indicating the ionic current reaches a maximum limitation of penetration depth [36–39].

To further illuminate Li^+^ storage capacity of SiO at different current densities, Fig. 5c shows the relationship between areal capacity and areal current density of LHGF/SiO electrodes at different mass loadings. Notably, the electrode with a mass loading of 94 mg cm^−2^ reaches an unprecedented areal capacity of 140.8 mAh cm^−2^, which is much higher than those of previously reported anode materials [20–25]. The curve shape is sigmoidal for every mass loading level. The areal capacity tends to saturate at a maximum value at low current density, and the electrodes with different mass loadings exhibit a quite sharp decrease in areal capacity when the current density exceed ~ 10–20 mA cm^−2^, indicating that the charge delivery is the main limiting factor at such high current density [38]. These analyses suggest that the charge transport is a major limiting factor to realize Li^+^ storage at ultra-high current density. In other words, without sufficient efficient delivery of the ion current, the active material cannot be fully utilized for Li^+^ storage. Furthermore, we have compared the areal capacity with the commercial graphite anodes and other representative anodes (such as Si and SnO_2_) at different current densities (Fig. 5d) [1, 3, 12, 33–36]. With an ultra-high intrinsic capacity and an unprecedented high mass loading, the optimized LHGF/SiO-75% electrode displayed an ultra-high areal capacity when compared with other state-of-art research and commercial devices.

Together, the LHGF/SiO-75% composite without additional current collector, conductive additive and binder, presents considerably higher areal capacity than that of other Si-based electrodes normalized by the total mass of the electrodes (Table 1). Moreover, the advantage of freestanding electrodes becomes more apparent when the mass of the inactive components (such as current collectors ~ 10 mg cm^−2^) are taken into account. We have estimated the gravimetric energy density of our full cell to be 393 Wh kg^−1^ at 1 C. By comparison, this value is ~ 31% higher than that of the state of the art (< 300 Wh kg^−1^), demonstrating the superlative cell-level performance enabled by LHGF/SiO-75% (Fig. S12). When considering the mass of inactive components, the advantages of high-mass-loading electrodes become even more obvious because of much smaller mass fraction of passive components (Table S3). For example, assuming an area-dependent overhead of ~ 10 mg cm^−2^ (for the current collectors and separators), the capacity and current density of the electrode with mass loading of 94, 44, and 21 mg cm^−2^ will only be reduced by about 9.7%, 18.5%, and 32.3%, respectively. In comparison, the practical capacity of a lower mass loading electrode (~ 1 mg cm^−2^) will decrease by more than 90%. Therefore, the LHGF/SiO-75% electrode with 3D freestanding graphene scaffold offers a promising pathway for realizing the potential high-capacity materials for practical energy storage devices.

**Table 1:** Comparison of LHGF/SiO composite electrodes with other silicon-based electrodes

Electrode	Mass fraction (%)	Active materials (%)	Loading (mg cm^−2^)	Rate capacity (mAh cm^−2^) @ (mA cm^−2^)		Refs
**LHGF/SiO**	**75.0**	**100**	**21**	**21.8 (2.1)**	**10.8 (10.5)**	This work
	**75.0**	**100**	**44**	**51.6 (4.4)**	**35.4 (8.8)**	
	**75.0**	**100**	**94**	**89.1 (9.4)**	**9.7 (28.2)**	
SiMP@Gr	91	80	0.8–2.5	1.5 (1.7)		[2]
SiO_x_/C-CVD	68.2	80	1.5–2.0	1.5 (2.0)		[6]
Y-S Si/C	78.0	80	2.0–3.5	2.1 (1.2)		[9]
AMPSi@C	91.5	80	2.9	4.1 (1.2)		[12]
p-Si/C	82.7	70	2.1	3.0 (5.5)		[33]
Si@CNT/C	85	50	2.2	5.6 (0.5)		[36]
SiO_x_/C	70	90	3.5	1.9 (11.4)		[40]
Si/TiO_2_/C	67.2	70	1.0–1.5	1.3 (2.0)		[41]
d-SiO@vG	97.5	75	1.5	3.3 (0.2)		[42]
NL-Si@C	–	80	1.0	0.97 (0.2)		[43]
void@SiO_x_@C	68.3	80	1.0	0.7 (0.5)		[44]
mpSi-Y	90	80	1–2.0	2.2 (0.2)		[45]
HNCSi	90	60	1.9–2.0	3.16 (0.4)		[46]
Si/C	60	20	3.3	2.0 (12.6)		[47]

Bold indicates the excellent battery performance (better than that of other electrodes)

## Conclusions

In conclusion, a mechanically robust LHGF/SiO composite was designed as a free-standing, binder-free anode for high-performance LIBs. The fully interconnected 3D graphene network serves as excellent conductive skeleton for electron conduction, and hierarchical 3D porous structure within the LHGF/SiO provides an ideal architecture for electrolyte permeation and efficient ion transport. Additionally, the specifically designed and elaborately engineered LHGF ensures excellent mechanical flexibility/robustness to accommodate the large volume expansion of SiO and maintain physical integrity and electrical connectivity of the electrode structures during the repeated charge/discharge process, and thus ensuring robust battery operation at ultra-high mass loading. Importantly, an optimized LHGF/SiO-75% electrode with a mass loading of 94 mg cm^−2^ delivers an exceptional areal capacity of 140.8 mAh cm^−2^ under a high current density of 4.7 mA cm^−2^ and retains a high areal capacity of 10.3 mAh cm^−2^ even at an ultra-high current density of to 28.2 mA cm^−2^, greatly exceeding the typical values observed in the state-of-the-art commercial or search anode (~ 3.5 mA cm^−2^ and 4 mAh cm^−2^). This work marks a critical step towards capitalizing high-capacity alloy-type electrode materials for high density energy storage technologies.

## Supplementary Information

Below is the link to the electronic supplementary material.

Supplementary file 1 (PDF 1318 kb)
