# Supplementary material for: A Silicon Monoxide Lithium-Ion Battery Anode with Ultrahigh Areal Capacity
Source: Nanomicro Lett. 2022 Jan 25;14:50. doi: 10.1007/s40820-022-00790-z (PMC8789978; doi:10.1007/s40820-022-00790-z)
Supplement: Supplementary file 1 — (PDF 1318 kb) [file 40820_2022_790_MOESM1_ESM.pdf]

Supporting Information for

A Silicon Monoxide Lithium-Ion Battery Anode with Ultrahigh Areal Capacity

Jiang Zhong[1], Tao Wang[1], Lei Wang[1], Lele Peng[2], Shubin Fu[3], Meng Zhang[1], Jinhui Cao[1], Xiang Xu[3], Junfei Liang[4], Huilong Fei[1], Xidong Duan[1], Bingan Lu[1], Yiliu Wang[1], Jian Zhu[1, *], and Xiangfeng Duan[5]

[1]State Key Laboratory for Chemo/Biosensing and Chemometrics, College of Chemistry and Chemical Engineering, School of Physics and Electronics, Hunan Key Laboratory of Two-Dimensional Materials, Engineering Research Center of Advanced Catalysis of the Ministry of Education, Hunan University, Changsha 410082, P. R. China

[2]International Graduate School at Shenzhen, Tsinghua University, Shenzhen 518057, P. R. China

[3]Key Laboratory of Structures Dynamic Behavior and Control of the Ministry of Education, Key Laboratory of Smart Prevention and Mitigation of Civil Engineering Disasters of the Ministry of Industry and Information Technology, Harbin Institute of Technology, Harbin 150090, P. R. China

[4]School of Energy and Power Engineering, North University of China, Taiyuan 030051, P. R. China

[5]Department of Chemistry and Biochemistry, University of California, Los Angeles, California 90095, USA

*Corresponding author. E-mail: jzhu@hnu.edu.cn (Jian Zhu)

S1 Supplementary Note

S1.1 Estimation of the Porosity of Compressed Electrodes

The density of the silicon oxide is 2.13 g cm^{-3}, and we assumed the density of the graphene was 2.0 g cm^{-3} (since the density of a graphene is approximately 1.5-2.0 g cm^{-3}). The theoretical density of the composite is calculated as [S1]:

$$\rho_{cal.} = \frac{m_{composite}}{\frac{m_{SiO}}{\rho_{SiO}} + \frac{m_{graphene}}{\rho_{graphene}}} \quad \text{(S1)}$$

The estimation of the porosity of the composite electrodes (ϕ) is:

$$\phi = \frac{\rho_{cal.} - \rho_{exp.}}{\rho_{cal.}} \quad \text{(S2)}$$

S1.2 Impedance Theory for Pores According to the Transmission Line Model (TLM)

For non-faradaic and faradaic processes at porous electrodes, the overall impedance is expressed as Eqs. S3 and S4, respectively.

$$Z_{nonfaradaic} = \sqrt{\frac{R_{ion,L}}{j\,\omega C_{dl,A} \times 2\pi\, r}}\, coth \sqrt{R_{ion,L} \times j\,\omega C_{dl,A} \times 2\pi\, r}\; L \qquad [S3]$$

$$Z_{faradaic} = \sqrt{\frac{R_{ion,L} \times R_{ct,A}}{(1 + j\omega R_{ct,A} \times C_{dl,A}) \times 2\pi r}}\, coth \sqrt{\frac{R_{ion,L}(1 + j\omega R_{ct,A} \times C_{dl,A}) \times 2\pi r}{R_{ct,A}}}\, L \qquad [S4]$$

The limiting values of the real (Z'ω) as ω→0 in non-faradaic, and faradaic processes are shown by Eqs. S3 and S4, respectively.

$$Z'_{nonfaradaic,\omega \to 0} = \frac{R_{ion}}{3} \qquad [S5]$$

$$Z'_{faradaic,\omega \to 0} = \frac{R_{ion}}{3} + R_{ct} \qquad [S6]$$

where R_{ion} is the mobility of Li ions inside the porous electrodes. From these mathematical equations, R_{ion} can be expressed as shown in Eq. S7 and S8:

$$R_{ion} = R_{ion,L} \times \frac{L}{n} \qquad [S7]$$

$$R_{ion,L} = \frac{\rho}{\pi r^2} \qquad [S8]$$

where $R_{ion,L}$ is ionic resistance per unit pore length. In addition, R_{ct} is expressed as Eq. S9:

$$R_{ct} = \frac{R_{ct,A}}{2\pi r L} \qquad [S9]$$

where $R_{ct,A}$ is the charge-transfer resistance per unit electroactive surface area. Using this combination approach, we have succeeded in separating the individual electrochemical parameters and corresponding kinetic interpretation from temperature dependence [S1, S2].

S1.3 Relaxation Time Constant According to the Complex Impedance Theory

The impedance $Z(\omega)$ can be written the complex form:

$$Z(\omega) = Z'(\omega) + jZ''(\omega) \qquad [S10]$$

An approach by using the impedance data to consider the cell as a whole:

$$Z(\omega) = \frac{1}{j\omega \times C(\omega)} \tag{S11}$$

Eq. S10 and S11 lead to Eq. S12:

$$C(\omega) = \frac{1}{\omega \times (jZ^{'}(\omega) - Z^{''}(\omega))} = \frac{-(Z^{''}(\omega) + jZ^{'}(\omega))}{\omega |Z(\omega)|^2} \tag{S12}$$

It is to define:

$$C(\omega) = C^{'}(\omega) - jC^{''}(\omega) \tag{S13}$$

leading to:

$$C^{'}(\omega) = \frac{-Z^{''}(\omega)}{\omega |Z(\omega)|^2} \tag{S14}$$

$$C^{''}(\omega) = \frac{Z^{'}(\omega)}{\omega |Z(\omega)|^2} \tag{S15}$$

where $C^{'}(\omega)$ is the real part of the capacitance $C(\omega)$, $C^{''}(\omega)$ is the imaginary part of the capacitance $C(\omega)$. The low-frequency value of $C^{'}(\omega)$ corresponds to the cell that is measured during constant-current discharge. It corresponds to an energy dissipation by an irreversible process that can lead to a hysteresis. The $C^{''}(\omega)$ is utilized to characterize the distribution of double electrode layer capacitance responsiveness. This time constant has earlier been described as a dielectric relaxation time characteristic of the whole system [S3-S5].

S1.4 Capacitive Contribution in LHGF/SiO

The capacitive contribution from the electrodes were obtained by separating the diffusion controlled capacity from the capacitance-controlled capacity according to the following equation:

$$i = k_1 v + k_2 v^{\frac{1}{2}} \tag{S16}$$

where i is the total current response at a fixed potential (V) during the CV test, $k_1 v$ is the contribution from the surface controlled process, and $k_2 v^{1/2}$ is the contribution from the diffusion controlled process. Hence, the percentage of surface contribution is calculated by:

$$C = \frac{k_1 v}{i} \tag{S17}$$

The LHGF/SiO-75 electrode shows a surface contribution of 3% at 0.1 mV s^{-1}. Upon gradually increasing the scan rate to 0.2 and 0.5 mV s^{-1}, the surface contribution raises to 5% and 9%, respectively. The areal capacity of the diffusion-controlled process (height of the red columns in Fig. 4d) decreases with the increases of the scan rate due to diffusion limitations. The CV characteristics suggest that the diffusion

contribution determined from the voltammetric-sweep-rate-dependence method is primarily faradaic process in our materials [S12].

S2 Supplementary Figures and Tables

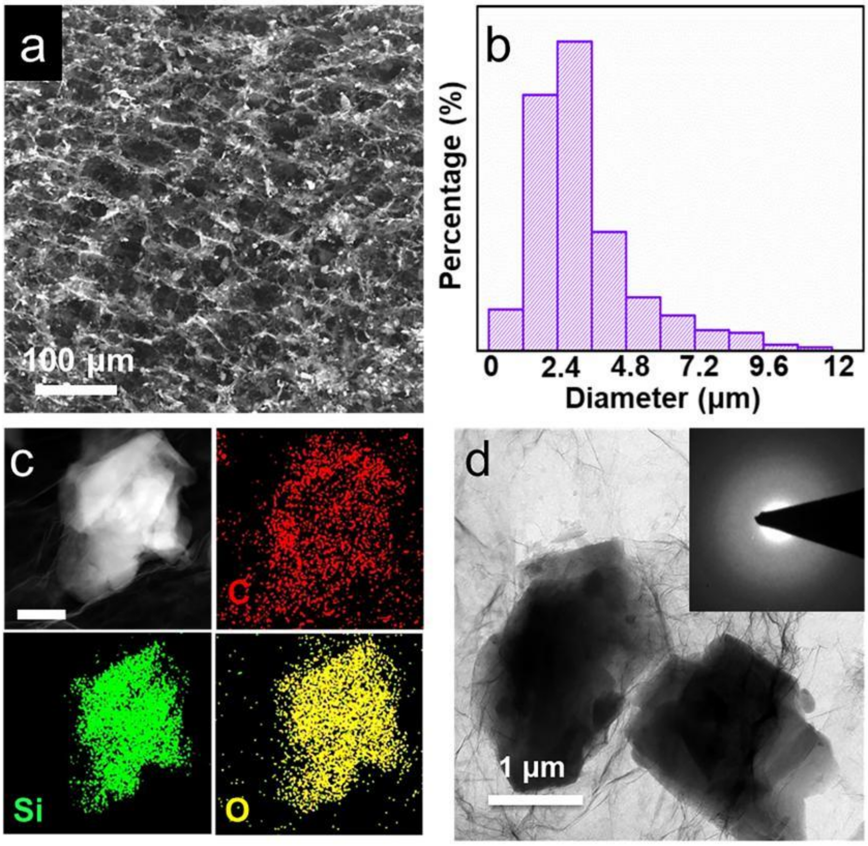

Fig. S1 a SEM images of 3D composite. **b** Diameter distribution of LHGF/SiO composite. **c** TEM image and corresponding EDS mappings of LHGF/SiO composite. Scale bar, 500 nm. **d** TEM and HRTEM image of SiO particles with uniformly decorated by graphene sheets

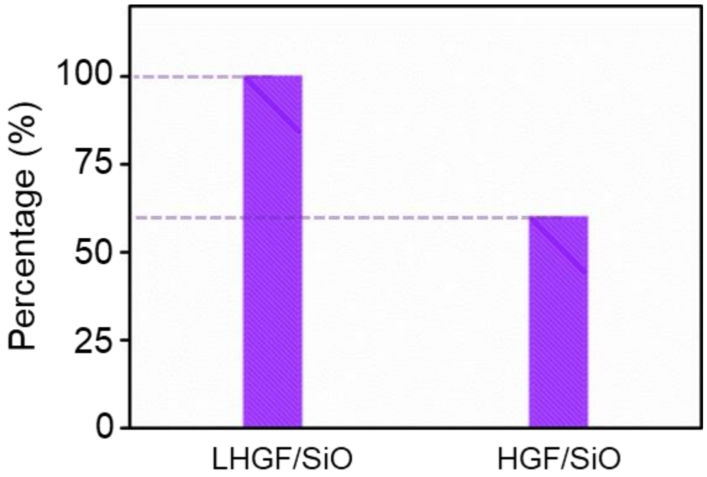

Fig. S2 The normalization processing of the tensile strength test for LHGF/SiO and HGF/SiO

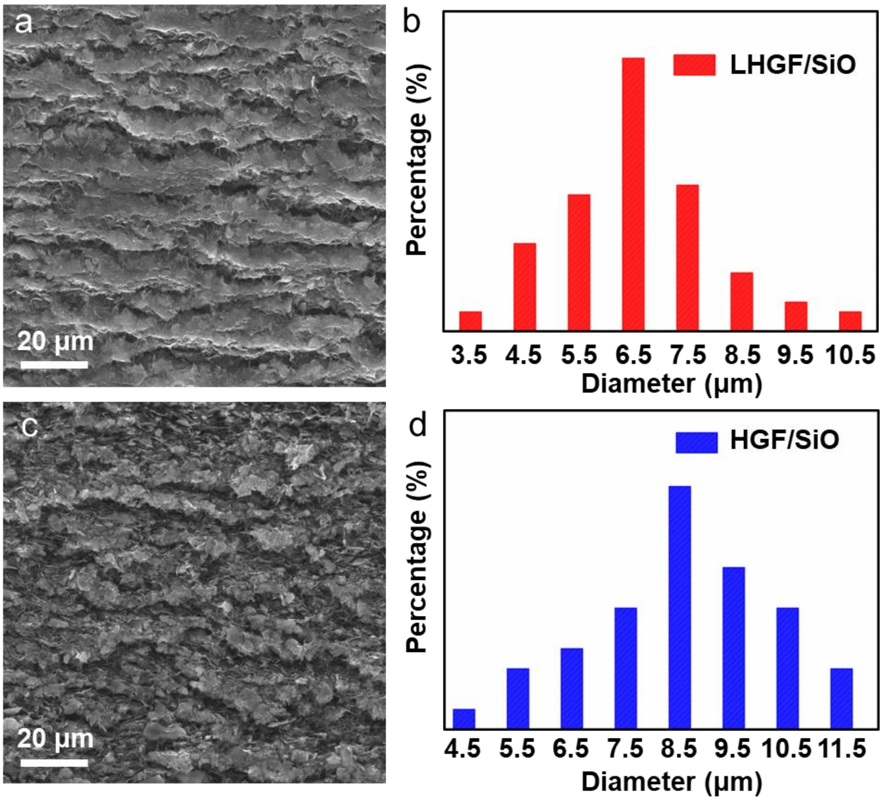

Fig. S3 a Cross-sectional SEM images of LHGF/SiO composite after discharge. **b** Diameter distribution of LHGF/SiO composite after discharge. **c** Cross-sectional SEM images of HGF/SiO composite after discharge. **d** Diameter distribution of HGF/SiO composite after discharge

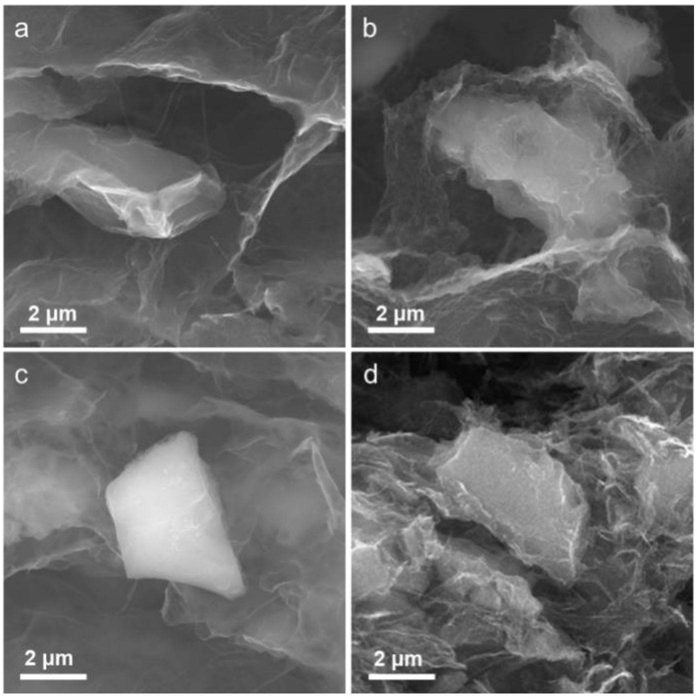

Fig. S4 a and **b** Cross-sectional SEM images of LHGF/SiO composite along vertical cuts before **a** and after **b** cycled. **c** and **d** Cross-sectional SEM images of HGF/SiO composite along vertical cuts before **c** and after **d** cycled

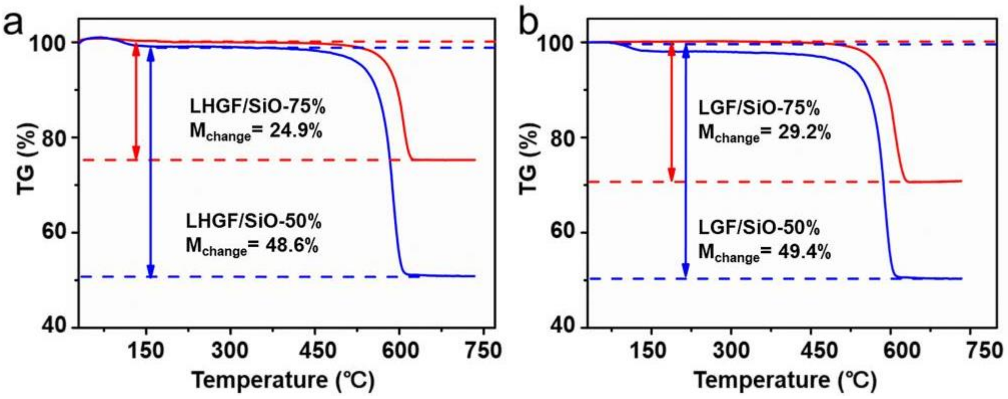

Fig. S5 a Thermogravimetric analysis of LHGF/SiO-50% and LHGF/SiO-75%. **b** Thermogravimetric analysis of LGF/SiO-50% and LGF/SiO-75% composites

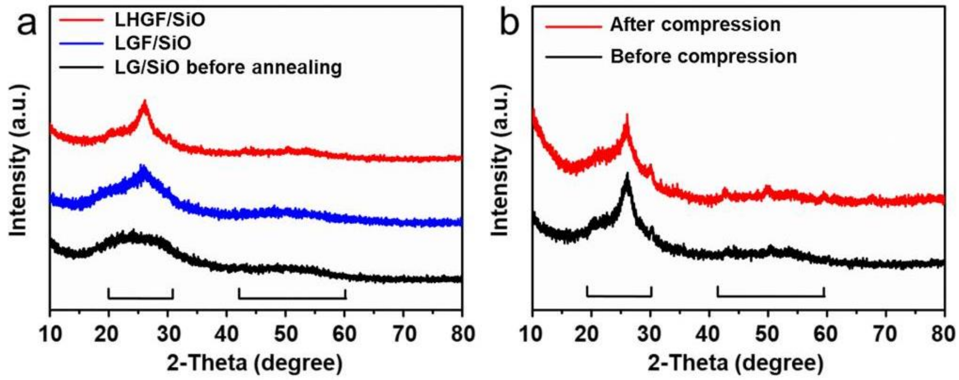

Fig. S6 a XRD patterns of LHGF/SiO, LGF/SiO and G/SiO composites. **b** XRD patterns of LHGF/SiO before and after mechanical compression

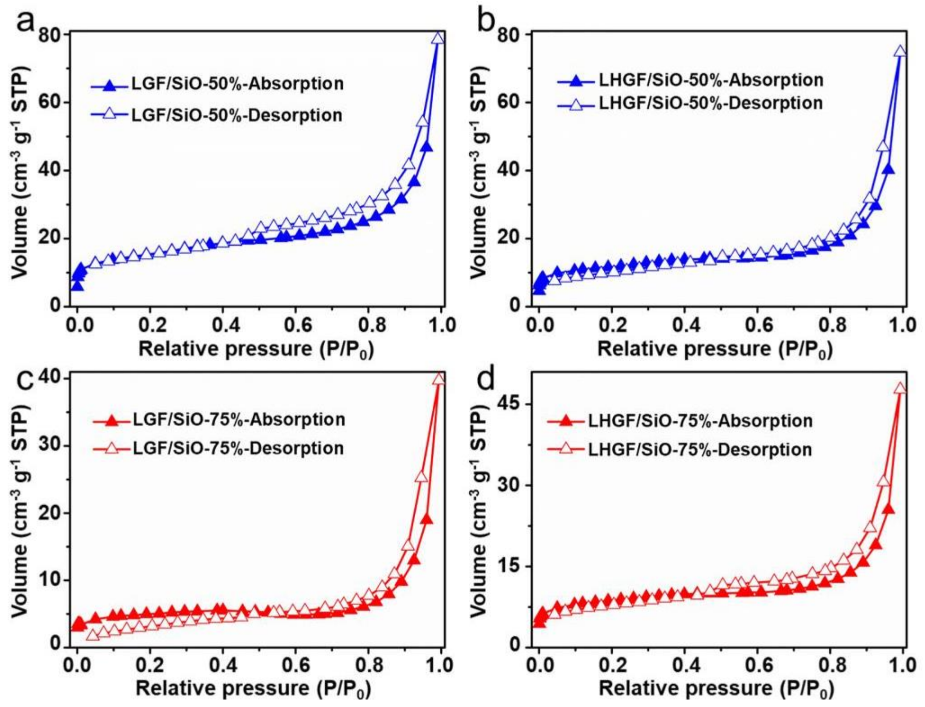

Fig. S7 a-d Nitrogen adsorption-desorption isotherms for LHGF/SiO and LGF/SiO composites

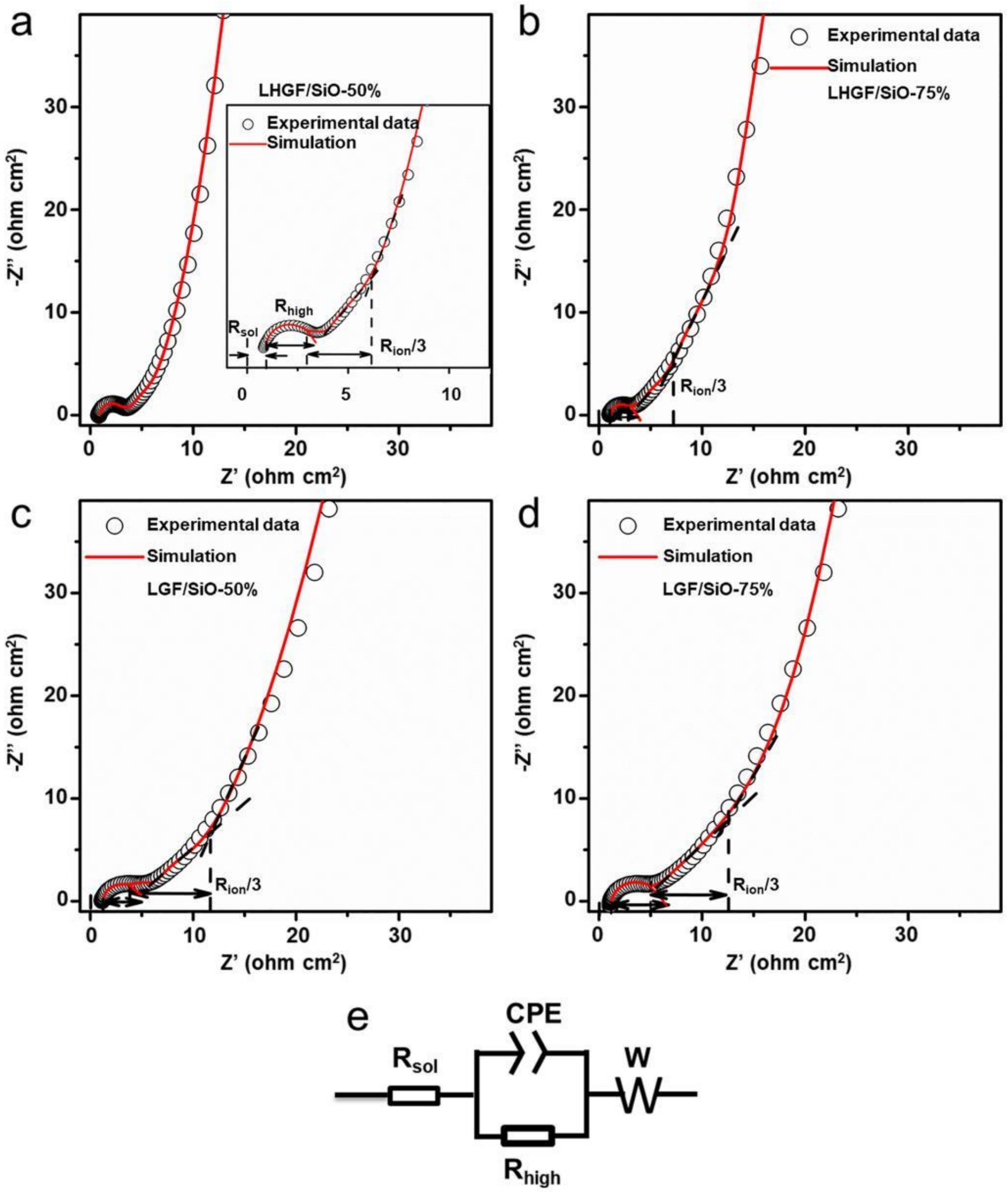

Fig. S8 a to **d** Nyquist plots for four types composites by using a symmetric cell with two identical electrodes (11 mg cm^{-2}) at unlithiated state (SOC = 0%). The solid line is the best-fitting simulation for the equivalent circuit shown in **e**. The inset in a shows how $R_{ion}/3$ is determined

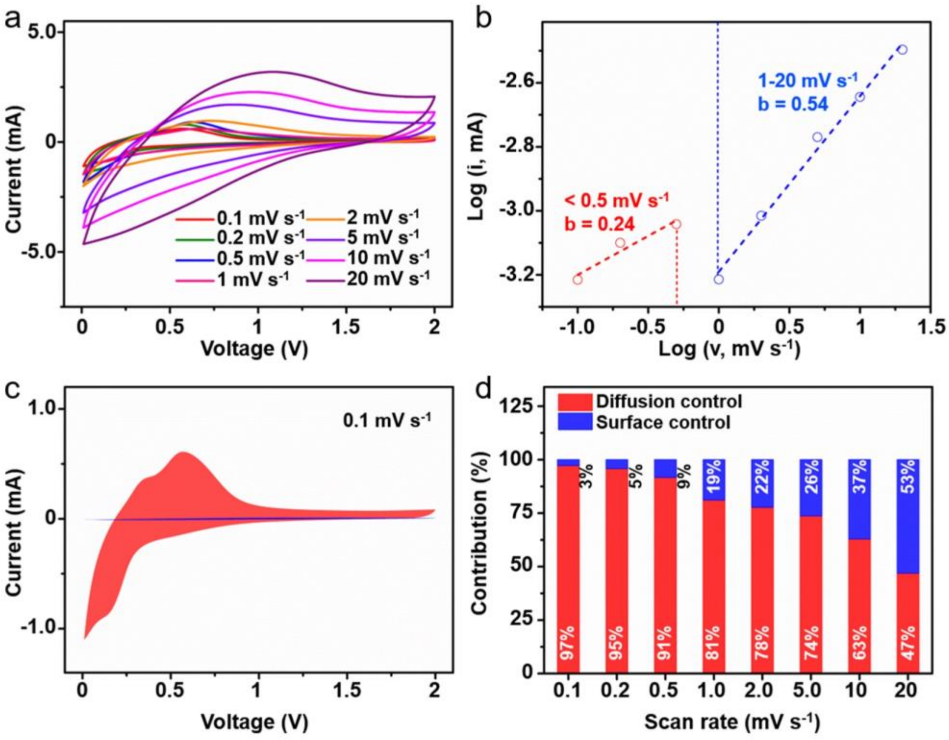

Fig. S9 Characterization of the charge storage mechanisms. **a** Cyclic voltammetry curves at various scan rates from 0.1 to 20 mV s^{-1}. **b** The relationship between peak current and scanning rate at low or high scanning rates. **c** Diffusion (red) and surface (blue) controlled contribution to Li^+ storage of LHGF/SiO-75% at 0.1 mV s^{-1}. **d** The proportion of diffusion controlled and surface controlled behavior under various scanning rates

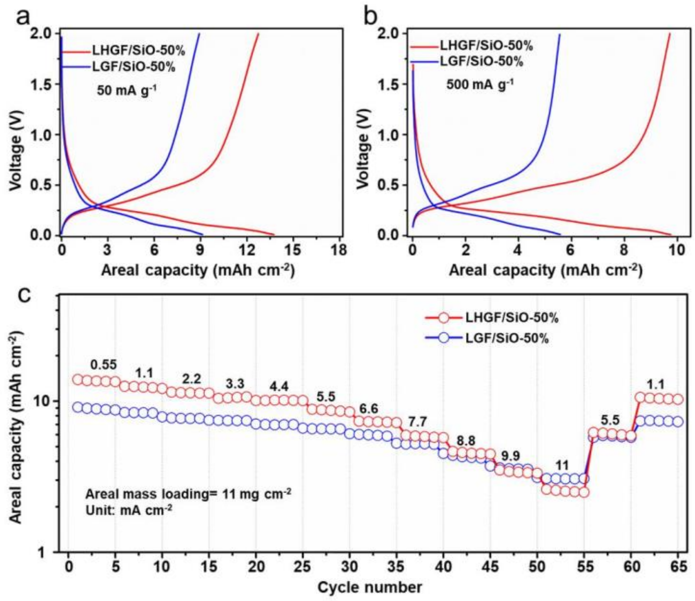

Fig. S10 a and **b**, Galvanostatic charge/discharge curves of two type electrode. **a** at 50 mA g^{-1} rate **b** at 500 mA g^{-1} rate. The mass loading is 11 mg cm^{-2}. **c** Rate-performances of LHGF/SiO-50% and LGF/SiO-50% electrode under same mass loading (11 mg cm^{-2})

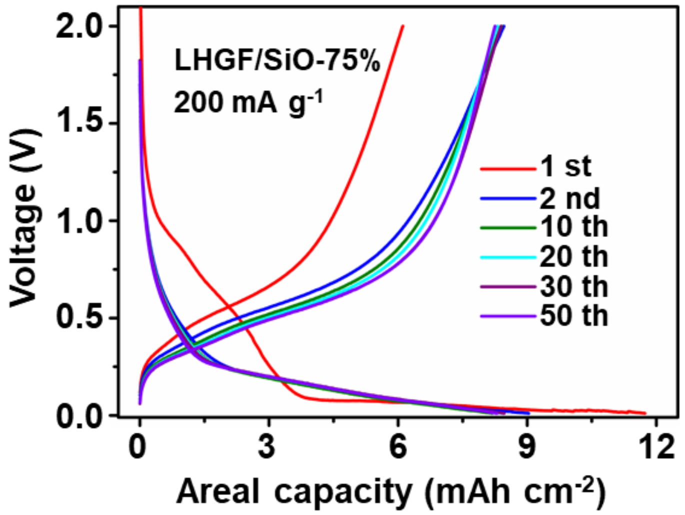

Fig. S11. The voltage versus specific capacity profiles of flexible LHGF/SiO-75% after 20 cycles of bending (A bending cycle is vertical fold)

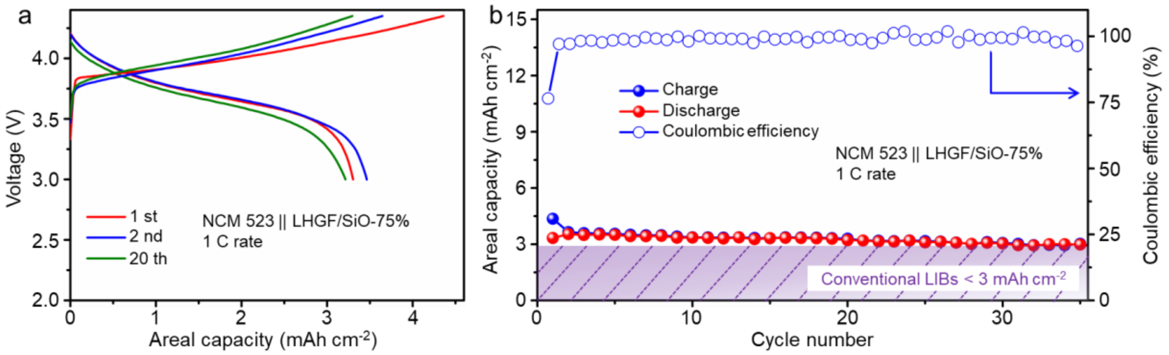

Fig. S12. a The charge/discharge voltage–capacity curves for full cells tested at 1 C rate. **b** Cycling performance of NCM 523‖LHGF/SiO-75% with the voltage range of 3-4.35 V. Commercial high-energy batteries typically have a maximum full-cell areal capacity cell of ~3 mAh cm^{-2}, as indicated by the violet hashed area

Table S1 Material parameters of various composites

Composites	V (cm^3 g^{-1})[1]	BET (m^2 g^{-1})	$V_{pore.}$ (cm^3 g^{-1})[2]	ϕ[2]	$\rho_{exp.}$ (g cm^{-3})[2]
LGF/SiO-50%	0.110	39	0.46±0.03	0.49±0.02	1.06±0.04
LHGF/SiO-50%	0.120	53			
LGF/SiO-75%	0.058	16	0.31±0.03	0.39±0.02	1.28±0.05
LHGF/SiO-75%	0.068	29			

Note: [1] The adsorption cumulative volume of pores between 1.7 and 300 nm width.
[2] Pore volume ($V_{pore.}$), porosity (ϕ) and the compacted density ($\rho_{exp.}$) of composite electrodes after compression were calculated based on 20 samples.

Table S2 Resistances determined from the transmission line model for symmetric cells with various porosities. The mass loading was 11 mg cm^{-2}

Electrode	R_{sol} (ohm cm^2)	R_{high} (ohm cm^2)	R_{ion} (ohm cm^2)
LGF/SiO-50%	1.13	3.82	23.61
LGF/SiO-75%	1.16	5.04	24.81
LHGF/SiO-50%	0.95	2.47	9.6
LHGF/SiO-75%	1.07	2.86	13.23

Table S3 Comparison of energy storage performance metrics for various Li-ion battery anode materials

	Electrode materials	Graphite	Micro-Si@G cages	SiO-PPy-500	SnO_2/HGF	YS-Si/C	AMPSi @C	**LHGF/SiO-75%**		
	Refs.	[S5]	[S6]	[S7]	[S8]	[S9]	[S10]	**This work**		
	Mass loading (mg cm^{-2})	12.5	0.8	1.75	12	2.83	2.9	**21**	**44**	**94**
Gravimetric	Capacity[1] (mAh g^{-1})	222	1260	1127.5	910	1272	1414	**1229**	**1277**	**951**
Gravimetric	Current density (mA g^{-1})	144	2100	100	100	200	414	**100**	**100**	**100**
Gravimetric	Capacity[2] (electrode+current collector) (mAh g^{-1})	124	102	168	500	281	318	**832**	**1041**	**859**
Gravimetric	Current density[2] (electrode+current collector) (mA g^{-1})	80	170	15	55	44	93	**68**	**82**	**90**
Gravimetric	Capacity (device)[3] (mAh g^{-1})	85	48	89	338	155	177	**625**	**874**	**782**
Gravimetric	Current density (device) (mA g^{-1})	55	80	8	37	25	52	**51**	**68**	**82**
Areal	Capacity (mAh cm^{-2})	2.8	1.1	1.97	11	3.6	4.1	**25.8**	**56.2**	**89.1**
Areal	Current density (mA cm^{-2})	1.8	1.68	0.175	1.2	0.02	1.2	**2.1**	**4.4**	**9.37**

Notes: [1] The gravimetric capacities are normalized by the total mass of electrodes including binders and carbon black; capacities in brackets are normalized by the active material only. [2] Assuming passive components (metal current collector and the separator) are about 10 mg cm^{-2}.[3] Calculated based on the industry standard (active materials account for 33% of total weight of the package when assuming the mass loading of the active material is about 10 mg cm^{-2}) [S5, S11].

Supplementary References

[S1] H. Sun, L. Mei, J. Liang, Z. Zhao, C. Lee et al., Three-dimensional holey-graphene-niobia composite architectures for ultrahigh-rate energy storage. Science **356**(6338), 599-604 (2017). https://doi.org/10.1126/science.aam5852

[S2] A.J. Bard, L.R. Faulkner, *Electrochemical methods: Fundamentals and Applications*. Wiley (1980).

[S3] N. Ogihara, Y. Itou, T. Sasaki, Y. Takeuchi, Impedance spectroscopy characterization of porous electrodes under different electrode thickness using a symmetric cell for high-performance lithium-ion batteries. J. Phy. Chem. C **119**(9), 4612–4619 (2015). https://doi.org/10.1021/jp512564f

[S4] P.L. Taberna, P. Simon, J.F. Fauvarque, Electrochemical characteristics and impedance spectroscopy studies of carbon-carbon supercapacitors. J. Electrochem. Soc. **150**, A292-A300 (2003). https://doi.org/10.1149/1.1543948

[S5] K.G. Gallagher, S.E. Trask, C. Bauer, T. Woehrle, S.F. Lex et al., Optimizing areal capacities through understanding the limitations of lithium-ion electrodes. J. Electrochem. Soc. **163**, A138–A149 (2015). https://doi.org/10.1149/2.0321602jes

[S6] Y. Li, K. Yan, H. Lee, Z. Lu, N. Liu et al., Growth of conformal graphene cages on micrometre-sized silicon particles as stable battery anodes. Nat. Energy **1**, 15029 (2016). https://doi.org/10.1038/nenergy.2015.29

[S7] S. Fang, N. Li, T. Zheng, Y. Fu, X. Song et al., Highly graphitized carbon coating on SiO with a π-π stacking precursor polymer for high performance lithium-ion batteries. Polymers **10**(6), 610 (2018). https://doi.org/10.3390/polym10060610

[S8] J. Liang, H. Sun, Z. Zhao, Y. Wang, Z. Feng et al., Ultra-high areal capacity realized in three-dimensional holey graphene/SnO_2 composite anodes. iScience **19**, 728-736 (2019). https://doi.org/10.1016/j.isci.2019.08.025

[S9] L. Zhang, C. Wang, Y. Dou, N. Cheng, D. Cui et al., A unique yolk-shell structured silicon anode with superior conductivity and high tap density for full Li-ion batteries. Angew. Chem. Int. Ed. **58**(26), 8824-8828 (2019). https://doi.org/10.1002/anie.201903709

[S10] W. An, B. Gao, S. Mei, B. Xiang, J. Fu et al., Scalable synthesis of ant-nest-like bulk porous silicon for high-performance lithium-ion battery anodes. Nat. Commun. **10**, 1447 (2019). https://doi.org/10.1038/s41467-019-09510-5

[S11] Y. Gogotsi, P. Simon, True performance metrics in electrochemical energy storage. Science **334**(6058), 917–918 (2021). https://doi.org/10.1126/science.1213003
